# Cross cultural adaptation and validation of burn specific health scale- brief in Nepali (BSHS-B-Np)

**DOI:** 10.1186/s41687-020-00190-0

**Published:** 2020-04-22

**Authors:** Regan Shakya, Misu Manandhar, Roshan Dangol, Archana Shrestha

**Affiliations:** 1 Department of Physiotherapy, Kathmandu University School of Medical Sciences/ Dhulikhel Hospital, Dhulikhel, Nepal; 2 Department of Physiotherapy, Sushma Koirala Memorial Hospital, Kathmandu, Nepal; 3 Department of Community Programs, Kathmandu University School of Medical Sciences/ Dhulikhel Hospital, Dhulikhel, Nepal

**Keywords:** Burns, Quality of life, Burn specific health scale, Questionnaire

## Abstract

**Background:**

Burns are a global health problem affecting the survivors and disrupting many aspects of their lives. It is the second most common injury in rural Nepal accounting 5% of disabilities. Burn Specific Health Scale (BSHS) is a valid and most commonly used tool to measure Health Related Quality of Life (HRQoL) of the patient with Burns. BSHS- B (Brief) has been translated, culturally adapted and validated in multiple languages but not in Nepali. Therefore we aim to translate, culturally adapt and validate the BSHS-B in Nepali language (BSHS-B-Np).

**Methods:**

Standard guideline was followed to translate the scale into Nepali language. One hundred eleven participants were evaluated to establish the psychometric properties of BSHS-B-Np. Internal consistency, test retest, content validity, discriminant validity and construct validity were assessed using Cronbach’s alpha, Interclass correlation coefficient, Factor analysis, Spearman rank test, and Mann- Whitney U test respectively.

**Results:**

The Cronbach’s alpha for BSHS-B-Np was 0.93. Test retest inter-class correlation coefficient was between 0.92 and 0.98. The principal component factor analysis with varimax rotation resulted in separation of nine factors explaining 75.19% of total variance. BSHS-B-Np showed good discriminant validity in 35 out of 36 domain correlations confirming the construct of the scale. Furthermore, the scale was able to discriminate between face, upper limb and lower limb injury (*p* < 0.05).

**Conclusions:**

BSHS-B-Np is a reliable and valid scale for Nepali burns survivors to assess their health related quality of life.

## Background

Burns are a global health problem affecting the survivors long after the injury and disrupting many aspects of patients’ lives [1, 2]. The majority of the estimated 180,000 deaths due to burns occur in low- and middle- income countries. It is the second most common injury in rural Nepal accounting 5% of disabilities [3]. In Nepal based on extrapolation, 557,032 people have suffered a burn injury with potentially 113,470 unable to receive appropriate surgical care [4].

The spectrum of burn trauma consists of minor injuries to disabling injuries. It affects relatively all aspects of person’s life in general and Health Related Quality of Life (HRQoL) in specific. Increasing complication and complexity of rehabilitation in burn victims make it essential for routine HRQoL evaluation, recommended as early as within 2 weeks of hospital discharge [5].

It is imperative to have valid and reliable tools to assess the HRQoL in patients with burns. Burn Specific Health Scale (BSHS) is a valid, commonly used and specific tool to assess the HRQoL in the patient with burns [6, 7].

BSHS is present in three different versions; Burn Specific Health Scale-Abbreviated (BSHS-A) [8], Burn Specific Health Scale- Brief (BSHS-B) [9] and the Burn Specific Health Scale Revised (BSHS-R) [10]. The BSHS-B is a 40 items a shortened scale with clinically meaningful factor domains improving the clinical use of the scale [7]. The scale has been translated, culturally adapted and validated in multiple languages [11–16]. Currently there is no validated Nepali version of BSHS-B available. Therefore we aim to translate, culturally adapt and validate the BSHS-B in Nepali language (BSHS-B-Np).

## Methods

This study was conducted in two steps. First, we translated and adapted BSHS-B English scale into Nepali; then it was validated using a cross-sectional study.

Outcome tool: BSHS- B is a scale measuring condition-specific health status among patients with burn injuries. It has 40 items across nine domains. The domains are Hand functioning, Simple abilities, Affect, Work, Sexuality, Interpersonal relationships, Body image, Heat sensitivity, Treatment regimen. The domains of hand functioning and simple abilities are categorized as Physical function domain. The domains of heat sensitivity and treatment regimen are categorized as non-physical/ burn specific domain. The other remaining five domains are categorized as social & economic function domain. Each item is rated in five point likert scale with 0: ‘Extremely’ to 4: ‘Not/none at all’ with the total score of 160. The score with a higher value indicates better HRQoL [14] and score 146/160 or above indicates adequate recovery [17].

### Translation of BSHS-B

The translation and adaptation was carried out with accepted procedure and standards [18]. First, two independent translators, an experienced cardiac surgeon fluent in English (medical background) and a non-medical professional translator did the forward translation. The non medical professional translator was not aware of the concepts in the scale. Both the translations were synthesized into a common version in consensus meeting between researchers and the translators. A psychiatrist was consulted for clarification of some items of the affect domain during the synthesis. Then two professionals, a qualified nurse fluent in English and non- medical professional translated the synthesized Nepali version to English. All of the translations were reviewed by an expert committee comprised of group of experts that included the translators, researchers, an experienced nurse of related practice, two senior physiotherapists and two non- health professionals. We encountered cultural irrelevance in translating two items; “I often feel sad and blue” and “getting in and out of chair”. The expert committee addressed the related issues and other minor translation issues to bring out the culturally adapted final Nepali version of the scale. (See additional file 1).

### Pretesting of BSHS-B-Np

Twenty patients with burn were provided BSHS-B- Np scale to complete and correspondingly rate the scale to assess its feasibility, understanding and clarity. We interviewed the participants in regard to their response wherein most of them had no problems with the scale. However two responders had difficulty in understanding the item “I have feeling of being trapped or caught”. We discussed this with a committee of three members (Surgery ward in-charge nurse, physiotherapist and a patient attendant) and decided to keep the original sentence. The participants took on an average of 20 minutes to complete the scale.

### Validation of BSHS-B-Np

#### Study setting and participants

We recruited a purposive sample of 111 patients with burns aged 18 years or older from two centers (Sushma Koirala Memorial Hospital, Kathmandu and Dhulikhel Hospital- Kathmandu University Hospital, Dhulikhel) from December 2016 to May 2018. Exclusion criteria were: (a) those less than 3 months since injury; (b) not fluent in Nepali; or (c) diagnosed psychiatric illness, chronic diseases, neurological disabilities, history of other trauma.

#### Participants’ recruitment

The eligible participants were enrolled by two methods; (a) those whose data were registered in hospital database were approached via telephone, (b) those who visited the hospital for the follow up treatment were approached in-person. We informed the participants about the study procedure and obtained written consent.

#### Data collection

We interviewed the participants using a standard questionnaire to collect the demographic and medical information such as age (in years), gender (male/ female), education level (<=10 standard/ > 10 standard), time since injury (in months), site of burn (Face& head, upper limb, lower limb, trunk, breast), thickness of burn (superficial, partial, full, deep bone), length of hospital stay (<=14 days/ > 14 days) and total body surface area of burn (%). In addition, we administered the BSHS-B-Np at this time and 2 weeks later [19]. Thirty six participants responded the second time.

#### Statistical analysis

The sample characteristics were summarized using mean (standard deviation) for continuous variables and frequency (percentage) for categorical variable. We assessed the test retest reliability for all the sub-domains and the total score of BSHS-B-Np using inter-class correlation coefficient (ICC) with two way mixed effects model. We used Kolgomorov- Smirnov’s (K-S) normal distribution test to determine the distribution of the test score, the statistically significant results indicated a non-uniform distribution. The internal consistency was determined for BSHS-B-Np using Cronbach’s alpha of each domain and total score. The principal components analysis was done with varimax rotation to compute the factor structure of items of BSHS-B-Np by extracting factors with eigen values greater than 1. Spearman’s rank correlation coefficient was used to compare the scores between each domain to determine the discriminant validity. We compared the scores between the group according to the site of injury (face and head, upper limb, upper limb) to establish the construct validity for BSHS-B- Np using Mann-Whitney U test.

## Results

### Sample description

The mean age of the participants was 29 (SD = 11) years. Forty two percent were male and the mean duration after injury was 40 (SD = 81) months. The mean total affected body surface area was 16 (SD = 8) percent. The majority of the sample had upper limb injuries (78%), lower limb (49%), facial & head (40%), trunk (39%), breast (24%). The sample consisted of 78% partial thickness injury, 17% full thickness, 3% superficial, 2% deep injury. More than half the participants (57%) had hospital stays of less than 14 days (Table 1).

**Table 1:** Baseline demographics of the study participants. (*n* = 111)

Characteristics	Frequency (%)
Age in years, Mean (SD)	28.9 ± 10.6
Gender	
Male	47 (42.3%)
Female	64 (57.7%)
Education level	
< =10 standard	73 (65.8%)
> 10 standard	38 (34.2%)
Time of injury (Months)	39.9 ± 81.2
Site of burn	
Facial & head burn	44 (39.6%)
Upper limb	86 (77.5%)
Lower limb	55 (49.5%)
Trunk	43 (38.7%)
Breast	27 (24.3%)
Thickness of burn	
Superficial	3 (2.7%)
Partial	87 (78.4%)
Full	19 (17.1%)
Deep	2 (1.8%)
Length of Hospital stay	
14 days or less	63 (56.8%)
More than 14 days	48 (43.2%)
Total Body Surface Area (%), mean ± SD)	16.6 ± 8.3

### Calculation of BSHS-B-Np

The total score range of BSHS-B- Np can be [0–160] with higher score indicating higher HRQoL. We observed the range of [37–157] with an average of 108.86 (SD 28). Each domain total score was calculated and results are shown in Table 2. To bring about the each domain values within the range of [0–4], we divided the total domain score with the total number of items in that domain. All observed all our average values are ranged [0–4] with highest mean observed in interpersonal relationship domain and least mean in work domain (Table 2).

**Table 2:** Means Standard deviation and Internal Consistency of BSHS-B- Np

BSHS-B- Np Domains	Items [Possible score range]	Mean (SD) [Observed score Range]		Cronbach’s alpha
		Sum of items	Average of items	
Heat sensitivity	5 [0–20]	11.16 (6.1) [0–20]	2.23 (1.2) [0–4]	0.89
Affect	7 [0–28]	20.70 (7.0) [3–28]	2.96 (1.0) [0–4]	0.87
Hand Function	5 [0–20]	14.46 (7.2) [0–20]	2.89 (1.4) [0–4]	0.96
Treatment regimens	5 [0–20]	14.10 (5.6) [0–20]	2.81 (1.1) [0–4]	0.87
Work	4 [0–16]	6.77 (5.1) [0–16]	1.69 (1.3) [0–4]	0.90
Sexuality	3 [0–12]	10.58 (2.8) [0–12]	3.53 (0.9) [0–4]	0.86
Interpersonal relationship	4 [0–16]	14.51 (3.2) [0–16]	3.63 (0.8) [0–4]	0.89
Simple Abilities	3 [0–12]	8.16 (4.2) [0–12]	2.72 (1.4) [0–4]	0.87
Body image	4 [0–16]	8.41 (5.3) [0–16]	2.10 (1.3) [0–4]	0.89
Total Score	40 [0–160]	108.86 (28.16) [37–157]	2.72 (0.7) [0–3.9]	0.93

### Reliability

#### Internal consistency

The internal consistency of every domain was above 0.86 which suggests a high level of homogeneity of the test. The cronbach’s alpha for BSHS-B-Np was 0.93 (Table 2).

#### Test retest reliability

The inter-class correlation coefficient score was excellent i.e. ranging above 0.8 for seven out of nine domains. The domain of sexuality had the minimum score of 0.48. The ICC score for the scale was 0.96 (Table 3).

**Table 3:** Test re-test reliability of BSHS-B- Np

Domains	Interclass correlation coefficient (ICC)	95% confidence interval		*P* value
		Lower bound	Upper bound	
Heat sensitivity	0.87	0.75	0.93	< 0.001
Affect	0.87	0.74	0.93	< 0.001
Hand Function	0.91	0.83	0.96	< 0.001
Treatment regimens	0.86	0.74	0.93	< 0.001
Work	0.82	0.65	0.91	< 0.001
Sexuality	0.48	−0.08	0.72	0.04
Interpersonal relationship	1	1	1	< 0.001
Simple Abilities	0.79	0.58	0.89	< 0.001
Body image	0.8	0.62	0.9	< 0.001
Total score	0.96	0.92	0.98	< 0.001

#### Factor analysis

The principal component analysis with varimax rotation resulted in separation of nine factors explaining 75.19% of total variance. Coefficients correspondent to the domain < 0.4 for the items were not shown in the table. Two items of simple ability (item 34 & item 35) corresponded additionally in the hand function domain with weak coefficient of less than 0.5. The analysis confirmed nine domains of BSHS-B-Np. In general, the result is similar to the findings of the original English version of the scale (Table 4).

**Table 4:** Rotated component matrix resultant from the Principal component analysis after Varimax with Kaiser Normalization

Factors		1	2	3	4	5	6	7	8	9
Eigen value		4.9	4.2	3.6	3.3	3.2	3.2	3.1	2.7	1.8
% total scale variance		12.3	10.5	9.0	8.2	8.1	8.1	7.7	6.7	4.6
Domains	Items									
Hand function	13	.89								
	14	.92								
	15	.91								
	16	.93								
	17	.84								
Affect	6		.77							
	7		.78							
	8		.79							
	9		.76							
	10		.73							
	11		.61							
	12		.61							
Heat sensitivity	1			.77						
	2			.82						
	3			.80						
	4			.82						
	5			.70						
Work	23				.77					
	24				.84					
	25				.83					
	26				.75					
Treatment regimens	18					.59				
	19					.76				
	20					.81				
	21					.82				
	22					.69				
Interpersonal relationships	30						.87			
	31						.86			
	32						.90			
	33						.71			
Body image	37							.87		
	38							.85		
	39							.79		
	40							.70		
Sexuality	27								.87	
	28								.88	
	29								.77	
Simple abilities	34	.49								.62
	35	.48								.61
	36									.69

*Note: factor loading value > 0.4 have been considered as significant*

#### Construct validity

The Kolmogorov-Smirnov (K-S) test significantly showed that the scores for the seven out of nine domains except for heat sensitivity and work domain were non-normally distributed. The total BSHS-B-Np scores were also statistically significant for K-S test. Spearman rank correlation coefficient test was performed for all inter domains correlations whereas Pearson’s correlation coefficient test was only performed between the domain of heat sensitivity and work domain. The correlations range of − 0.5 to 0.5 between the domains indicates good discriminant validity [20]. We obtained 34 out of 36 correlations within the range indicating the construct validity of the scale. The correlation of between domain of simple ability with the domains of hand function and work was observed to be greater than 0.5 (Table 5). All the domains except for the interpersonal relations domain significantly correlated with total score of BSHS-B-Np. The Mann Whitney U test was performed between two non-related groups of scores of each domain and total BSHS-B-Np score. There was significant difference in scores of heat sensitivity, body image and social emotional domain while comparing the patients with and without injury to face and head; scores of hand function, sexuality and physical function domain comparing those with and without upper limb injury; and scores of hand function and physical function domain comparing those with and without lower limb injury (Table 6).

**Table 5:** Correlation between the domains of BSHS-B-Np

Domains	Affect	Hand Function	Treatment regimens	Work	Sexuality	Interpersonal relationship	Simple Abilities	Body image	Total BSHS
Heat sensitivity	0.4^**^	0.23^*^	0.31^**^	0.29^**^	0.1	−0.04	0.28^**^	0.34^**^	0.59^**^
Affect		0.06	0.44^**^	0.16	0.25^**^	0.2^*^	0.16	0.31^**^	0.57^**^
Hand Function			0.22^*^	0.42^**^	0.19^*^	0.13	0.57^**^	0.02	0.55^**^
Treatment regimens				0.41^**^	0.26^**^	0.11	0.36^**^	0.39^**^	0.65^**^
Work					0.12	−0.02	0.6^**^	0.37^**^	0.72^**^
Sexuality						0.36^**^	0.29^**^	0.06	0.32^**^
Interpersonal relationship							0.13	0.1	0.19
Simple Abilities								0.17	0.66^**^
Body image									0.55^**^

**Table 6:** Subgroup comparison of BSHS-B- Np

Domains	With Face & Head (*n* = 44)	Without Face & Head (*n* = 67)	P value*
Total score	103.3 ± 29.9	112.5 ± 26.6	0.12
Heat sensitivity	9.4 ± 6.0	12.3 ± 5.9	0.02
Body image	6.9 ± 4.8	9.4 ± 5.4	0.02
Social emotional domain	57.6 ± 15.9	63.2 ± 14.0	0.01
Non physical domain	22.6 ± 9.6	27.0 ± 9.3	0.05
	With Upper Limb (*n* = 86)	Without Upper Limb (*n* = 25)	
Total score	106.7 ± 29.8	106.3 ± 20.5	0.22
Hand Function	13.1 ± 7.6	19.2 ± 1.9	0.00
Sexuality	10.3 ± 3.1	11.6 ± 0.9	0.04
Physical function domain	21.2 ± 10.8	27.6 ± 5.2	0.04
	With Lower Limb (*n* = 55)	Without Lower Limb (*n* = 56)	
Total score	107.1 ± 29.2	110.6 ± 27.2	0.45
Hand Function	16.5 ± 5.7	12.4 ± 8.0	0.03
Physical function domain	25.0 ± 8.9	20.2 ± 10.9	0.03

*Mann Whitney U test

## Discussion

In this study, we translated, culturally adapted and validated the BSHS-B scale into the Nepali language. We inserted “Np” suffix to the scale acronym for identification as Nepali version: BSHS-B-Np. The result showed that the Nepali version scale is a reliable and valid tool which can be used in clinical research and practice. The scale showed excellent internal consistency with recommended cronbach’s alpha above 0.8 [21]. The coefficient of 0.93 for overall scale score reflected good homogeneity of the translation which is similar to finding of the original scale and similarly with other versions as well [9, 11, 22]. The high index of 0.96 was found for the domain of hand function. Another test to determine the reliability of the scale is the test retest reliability. We administered the scale a second time after 14 days for 36 participants and obtained the ICC of 0.96 (0.92–0.98) for the overall score. The value of hand function domain index of 0.91 showed excellent agreement. Similarly the domain of heat sensitivity, affect, treatment regimen, treatment regimens, work, body image and simple abilities demonstrated the value above 0.8 which showed good agreement. However the scale of sexuality demonstrated moderate agreement with the value of 0.48. The inconsistency in reporting sexually related behavior could have been influenced by many factors including socio-cultural norms, sexual health education, physical or psychological factors [23, 24]. Multiple studies have also reported tendency of participants with burns to underreport sexual problems and its consequences [12, 25, 26].

The sample size for principal component analysis with varimax rotation was poor but acceptable [27]. The total variance obtained was 75.19% with the eigen value kept greater than 1. The analysis created nine domains mirroring the original scale with each domain having higher load value on a common factor (> 0.4). Each domains resulted with good internal consistency can therefore be used as a separate subscale [9].

The correlation analysis was done between the domains created. We performed the KS normality test to determine the distribution of the sample obtained in each domain. We found the samples significantly didn’t distribute normally for all domains except for the domains of heat sensitivity and work. Therefore spearman’s rank correlation was used for non normal distribution and Pearson correlation coefficient was used for normally distributed samples. According to Carlson and Herdman convergent validity is claimed if the correlation coefficient is above 0.5 [28]. In our study the correlation coefficient between the obtained domains was below 0.5 for 34 out 36 correlations establishing the discriminant validity therefore confirming the construct validity of the scale. Correlations between simple ability domain with hand function and work domains were greater than 0.5 indicating the poor discriminant validity between the domains. The fact that simple tasks such as bathing and dressing requires hand functioning could explain the poor discriminant validity. Factor reports of additional weak coefficient between simple ability items (item 34 and item 35) in hand function domain indicate the positive correlation between them. Positive correlation between simple ability and work domains was also reported in the original paper [9]. Other versions researches have also demonstrated weak to moderate inter domain correlations [13, 20, 29].

To further confirm construct validity we assessed the burn injuries of the face and head, upper limb and lower limb. A valid scale produces the statistically significant difference between results of both groups. In this study the injury at the face and head discriminated significantly with heat sensitivity, body image, socio-emotional, non physical domain. Similar findings was observed in Hebrew version in which head and face discriminated with heat sensitivity, work, sexuality socio-economic, non-physical domain, BSHS total score [14]. It is well understood that the face is an important body part and facial injury influences the majority of the scale’s domains. In our study participants with burns injury at both upper and lower limb discriminated significantly with hand function and physical function domain. Similarly, we observed that upper limb discriminated with sexuality. This suggests that activities of performance are dependent on the function of extremities. Similar findings were also observed in other studies [14, 16].

### Strength and limitation

Our study’s strength includes the use of factor analysis to test the factor structure of the scale. The factor analysis created nine domains mirroring the original scale [9].

Our study isn’t devoid of limitations. The major limitation was the small sample size. This could be due to lack of burn rehabilitation centers in Nepal. The burn centers operating in Nepal chiefly provide services to acute and sub acute care for the burn victims. Subsequently, the rate of hospital visits decreases over period of time after the hospital discharge. Majority of our samples were burn survivors who visited hospital for the follow-up treatments. Similarly, it affected the retest samples as well since response rate after 2 weeks was poor which resulted in only 36 participants. Secondly, we didn’t use any marker of stability to identify unchanged patient over 2 week period which could have resulted in poorer retest on sexuality. Further studies with larger sample size are recommended.

## Conclusions

In conclusion, the Nepali version of BSHS-B is a valid and reliable scale for measuring the burn injury status of the Nepali people. It is culturally appropriate tools which can be used in clinical setting to determine the quality of life of Nepali burn survivors.

## Supplementary information

**Additional file 1.** Nepali Version of Burn Specific Health Scale- B- Np

## Data Availability

The dataset generated and/or analyzed during the current study are available from the corresponding author on a reasonable request.

## Abbreviations

HRQoL: Health Related Quality of Life
BSHS: Burn Specific Health Scale
BSHS-A: Burn Specific Health Scale- Abbreviated
BSHS-B: Burn specific Health Scale- Brief
BSHS-R: Burn Specific Health Scale- Revised
BSHS-B-Np: Burn Specific Health Scale- Brief- Nepali
ICC: Interclass correlation coefficient
KS test: Kolgomorov- Smirnov’s test
SD: Standard Deviation
OR: Odds Ratio
CI: Confidence Interval
SPSS: Statistical Package for the Social Sciences
