# Supplementary material for: Cross cultural adaptation and validation of burn specific health scale- brief in Nepali (BSHS-B-Np)
Source: J Patient Rep Outcomes. 2020 Apr 22;4:25. doi: 10.1186/s41687-020-00190-0 (PMC7176760; doi:10.1186/s41687-020-00190-0)
Supplement: Supplementary file 1 — Additional file 1. Nepali Version of Burn Specific Health Scale- B- Np [file 41687_2020_190_MOESM1_ESM.docx]

| Additional file 1: Nepali Version of Burn Specific Health Scale- B- Np  (**kf]n]sf] 3fpsf] ljlzi6 :jf:Yo dfkg – ;+lIfKt**) | | | | | |
| --- | --- | --- | --- | --- | --- |
|  | cToflws | Psbd | dWod | clnslt | 5}g, x'Fb}g |
| **tfksf] ;+j]bgzLntf** | | | | | |
| aflx/ 3fddf hfg dnfO{ emGem6 x'G5 . |  |  |  |  |  |
| udL{n] dnfO{ lk/f]N5 . |  |  |  |  |  |
| d udL{df aflx/ hfg / sfdx? ug{ ;lSbg . |  |  |  |  |  |
| d 3fddf aflx/ lg:sg ;lSbg eGg] s'/fn] dnfO{ lk/f]N5 . |  |  |  |  |  |
| d]/f] 5fnf klxnfsf] eGbf a9L ;Da]bgzLn ePsf] 5 . |  |  |  |  |  |
| **c;/** | | | | | |
| tkfO{nfO{ s'g xb;Dd j0f{g u5{: d k\|fo b'vL jf pbf; x'G5' . |  |  |  |  |  |
| s'g} a]nf d ;f]R5', dnfO{ efjgfTds ;d:of 5 . |  |  |  |  |  |
| tkfO{nfO{ s'g xb;Dd j0f{g u5{: PSnf]kgsf] cfef;n] dnfO{ ;tfPsf] 5 . |  |  |  |  |  |
| dnfO{ cNem]sf] jf kmGbfdf k/]sf] dx;'; x'G5 . |  |  |  |  |  |
| dnfO{ dfG5]x?;+u e]63f6 ug{ /dfOnf] nfUb}g . |  |  |  |  |  |
| d]/f] ;d:ofx?sf] af/]df s'/f ug{ d ;+u sf]lx 5}g . |  |  |  |  |  |
| dnfO{ ;fyLx?;+u s]lx ug{ ?lr 5}g . |  |  |  |  |  |
| **xftsf] sfd** | | | | | |
| tkfO{nfO{ slt sl7gfO{ 5 , x:tfIf/ ug{ . |  |  |  |  |  |
| tkfO{nfO{ slt sl7gfO{ 5 , xft jf rDrfn] vfg . |  |  |  |  |  |
| tkfO{nfO{ slt sl7gfO{ 5 , ;Dd k/]sf] ;txaf6 l;Ssfx? l6Kg. |  |  |  |  |  |
| tkfO{nfO{ slt sl7gfO{ 5 , 9f]sfsf] r's'n÷5]l:sg vf]Ng . |  |  |  |  |  |
| tkfO{nfO{ slt sl7gfO{ 5 , ;'?jfn, h'Qf cflbsf] t'gf afFWg . |  |  |  |  |  |
| **pkrf/ k4ltx?** | | | | | |
| d]/f] 5fnfsf] x]/rfx ug'{ Ps emGem6 xf] . |  |  |  |  |  |
| d]/f] kf]nfO{sf] nflu ug{ elgPsf s'/fx? 5g\ h''g dnfO{ ug{ dg nfUb}g . |  |  |  |  |  |
| d rfxG5' d]/f] kf]nfO{sf] :ofxf/sf] nflu d}n] olt w]/} s'/fx? ug{ gk/f];\ . |  |  |  |  |  |
| d]/f] kf]nfO{sf] :ofxf/sf] nflu ug{ elgPsf ;a} sfo{x? ug{ dnfO{ sl7g 5 . |  |  |  |  |  |
| kf]nfO{sf] x]/ljrf/ ubf{ d]/f cGo dxTjk"0f{ sfo{x? ug{ sl7g 5 . |  |  |  |  |  |
| **sfd** | | | | | |
| d]/f] kf]nfO{n] d]/f] sfddf afwf kf5{ . |  |  |  |  |  |
| kf]lnPsf] x'gfn] d]/f] sfo{ Ifdtfdf c;/ k/]sf] 5 . |  |  |  |  |  |
| kf]nfO{n] ubf{ d]/f] sfddf ;d:of k/]sf 5g\ . |  |  |  |  |  |
| tkfO{nfO{ slt sl7gfO{ 5 , k'/fgf] k]zf ug{ / k'/fgf] lhDd]jf/Lx? lgefpg] . |  |  |  |  |  |
| **of}g ;DalGw** | | | | | |
| cfkm" klxnf]h:tf] of}g pQ]lht x'g g;Sbf dnfO{ lbSs nfU5 . |  |  |  |  |  |
| dnfO{ ;fdfGo tyf zf/Ll/s ;DaGw /fVg ?lr 5}g . |  |  |  |  |  |
| d ca cFufnf] dfg{, xft ;dfTg jf r'Dag ug]{ ulb{gF . |  |  |  |  |  |
| **cGt/JolQm ;DaGwx?** | | | | | |
| d]/f] kl/jf/n] d]/f] jl/kl/ ug]{ Jojxf/ dnfO{ dg kb}{g . |  |  |  |  |  |
| d kl/jf/;+u x'g'eGbf a? PSn} x'g rfxG5' . |  |  |  |  |  |
| d gx'Fbf g} d]/f] kl/jf/nfO{ /fd\|f] x'GYof] . |  |  |  |  |  |
| d]/f] rf]6n] ubf{ dnfO{ d]/f] kl/jf/af6 cem} 6f9f /fv]sf] 5 . |  |  |  |  |  |
| **;fwf/0f Ifdtf** | | | | | |
| tkfO{nfO{ slt sl7gfO{ 5 , cfkm} g'xfpg . |  |  |  |  |  |
| tkfO{nfO{ slt sl7gfO{ 5 , cfkm} n'uf nufpg . |  |  |  |  |  |
| tkfO{nfO{ slt sl7gfO{ 5 , s';L{df a:g / p7\g . |  |  |  |  |  |
| **zf/Ll/s k\|ltlaDa** | | | | | |
| d]/f] vtx?sf] :j?kn] dnfO{ lk/f]N5 . |  |  |  |  |  |
| d]/f] ;fdfGo :j?kn] ;fFRr} g} dnfO{ lk/f]N5 . |  |  |  |  |  |
| slxn]sflx d d]/f] :j?k kl/jt{g ePsf] la;{g rfxG5' . |  |  |  |  |  |
| dnfO{ nfU5 ls d]/f] kf]nfO{sf] vt c?nfO{ cgfsif{s x'G5 . |  |  |  |  |  |
